# On creativity and meaning: The intricate relationship between creativity and meaning in life and creativity as the means to repay existential debt

**DOI:** 10.1080/19420889.2025.2484526

**Published:** 2025-03-30

**Authors:** Tobore Onojighofia Tobore

**Affiliations:** Independent Scholar, Yardely, USA

**Keywords:** Cancel culture, censorship, creativity, creativity decline, evolution, evolutionary debt, existential debt, freedom of expression, meaning, meaning crisis, meaning in life, meaning issues, mental health, modern societies, psychological well-being, stress, suicide, Existential Meaninglessness

## Abstract

Creativity, which is the leverage of imagination to attain valued goals, is one of the defining features of humans. It is the trait that gives an advantage to humans in solving problems, enhancing their survival. Creativity is a critical evolved trait, hard-wired in the human genome and linked with many benefits, including mating success, psychological well-being, and human thriving. Evidence suggests creativity is a critical source of meaning. Many features of the modern world promote the interrelated factors of low trust, fear, and acute stress which make people vulnerable to meaninglessness or meaning crisis and these same factors negatively impact creativity. This suggests a relationship between meaning in life and creativity in which meaninglessness may negatively impact creativity and vice versa. In this paper, the role of creativity in providing meaning in human life, as the essence of human existence to repay our evolutionary or existential debt, and the intricate relationship between psychological well being, creativity and meaning in life are discussed. The need and ways to prioritize creativity in society to improve psychological well-being and make people live meaningfully are also discussed.

## Creativity

Creativity is seen in many animal species and is one of the defining features of humans. It can be defined as the leverage of original ideas or imagination to attain valued goals [1]. It can also be defined as the human capability to use imagination to create novel and useful solutions for complex problems [2]. Unlike intelligence, which is critical to the fulfillment of the more basic needs, creativity is associated with higher human needs [3]. The earliest signs of hominid creativity are traced to the development of simple stone tools, believed to be made by Homo habilis, over two million years ago [4]. There is a long history of creativity from early hominids to modern humans with the evolution of cranial capacity [4]. Creativity is a critical evolved trait [5], hard-wired in the human genome [1,6]. Besides the genome, different neural transmitters, structures, and networks play a role in creativity, including gray matter volume, salience network, executive attention network, default network, prefrontal cortex, orbitofrontal cortex, right angular gyrus, etc [7–12]. Aneural cells have also been hypothesized to be capable of creativity [13].

Creativity confers significant biological benefits. Indeed, evidence suggests that sexual selection likely played a role in the evolution of creativity [14] and creativity is linked to mating success [15–17]. It confers an advantage in humans thriving [18] and subjective well-being [19,20]. Human creativity gave an advantage to earlier humans in solving problems enhancing their survival [21]. It is the foundation of human cultural evolution [22] and the trait that has made humans a truly cosmopolitan species, able to conquer any environment on Earth and in cosmic spaces [4,23].

Meaninglessness has been described as one of the biggest threats of our time [24] and many features of the modern world make people vulnerable to meaning crisis [25]. Indeed, the world, it is argued, is in the midst of an existential crisis [26]. The objective of this paper is to discuss the critical role of creativity in providing meaning in human life, its role in human progress, its function as the essence of human existence, and the intricate relationship between creativity and meaninglessness or meaning crisis.

## Components of creativity

**Figure 1.**
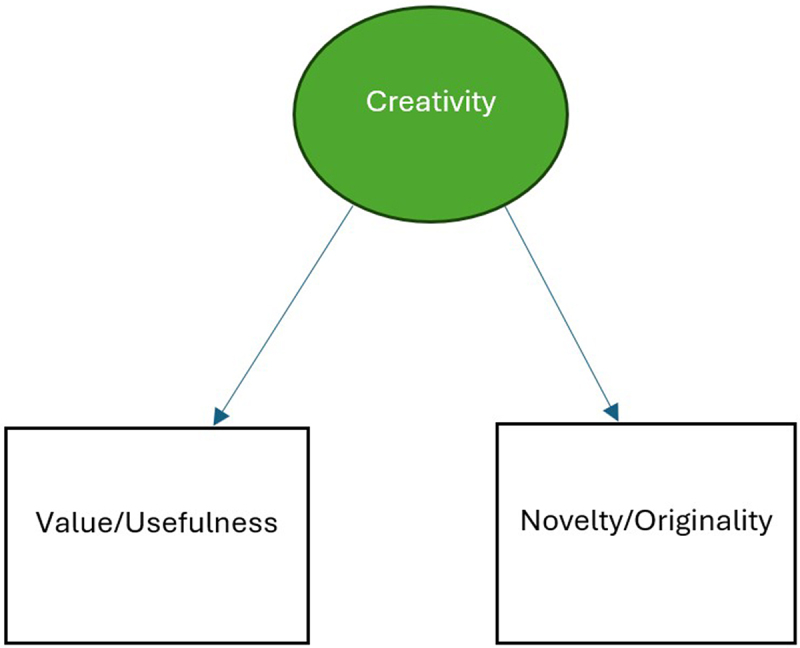
Main components of creativity.

Creativity, which is the leverage of original ideas or imagination to attain valued goals [1] has two critical components: originality or novelty and value or usefulness. Culture shapes people’s conceptions of creativity and how these two components are viewed. Indeed, in the conception of creativity in collectivist or Eastern cultures, value or usefulness appears more important than novelty, whereas novelty seems just as important as value, maybe even more in Western cultures [27,28]. Overwhelmingly, these two components are integral to the understanding of creativity (Figure 1).

**Figure 2.**
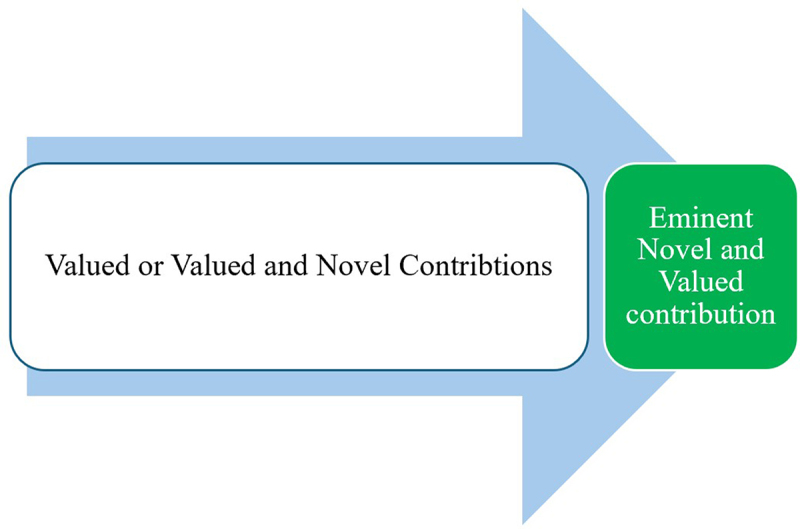
The spectrum of creative contributions.

A critical takeaway from the creativity components is that for any effort to meet the threshold of creativity, it must be novel and valuable. Novelty means that the idea, approach, or solution is new, original, or different. Value entails that it is of value to the owner, inventor, or originator and people in the environment. Furthermore, human creative contributions are in a spectrum with eminently valued and novel contributions at the end of the spectrum. Eminently valued and novel contributions are extraordinary and propel society forward. They change the world, moving it into the future. They are the foundation of human progress and the ultimate mitigating trump card against extinctionary forces (Figure 2).

## Creativity and meaning in life

Like creativity, meaning is essential to human thriving and well-being. Meaning in life, described as a state of perceiving one’s life as having coherence, and purpose plays an important role in every facet of human life including during good or bad times [29]. It is critical to psychological well-being [30]. Unsurprisingly, when people struggle with meaninglessness, this causes existential anxiety [31] which is correlated with increased stress, general anxiety, and depression [31]. Meaninglessness impacts psychopathology [32,33] and suicidal ideation and tendencies [34–36]. In contrast, meaning in life has a protective impact against suicide [35,36] and psychopathology [32] and is deeply connected to them through its association with hope [33].

### Creativity as a source of meaning

**Figure 3.**
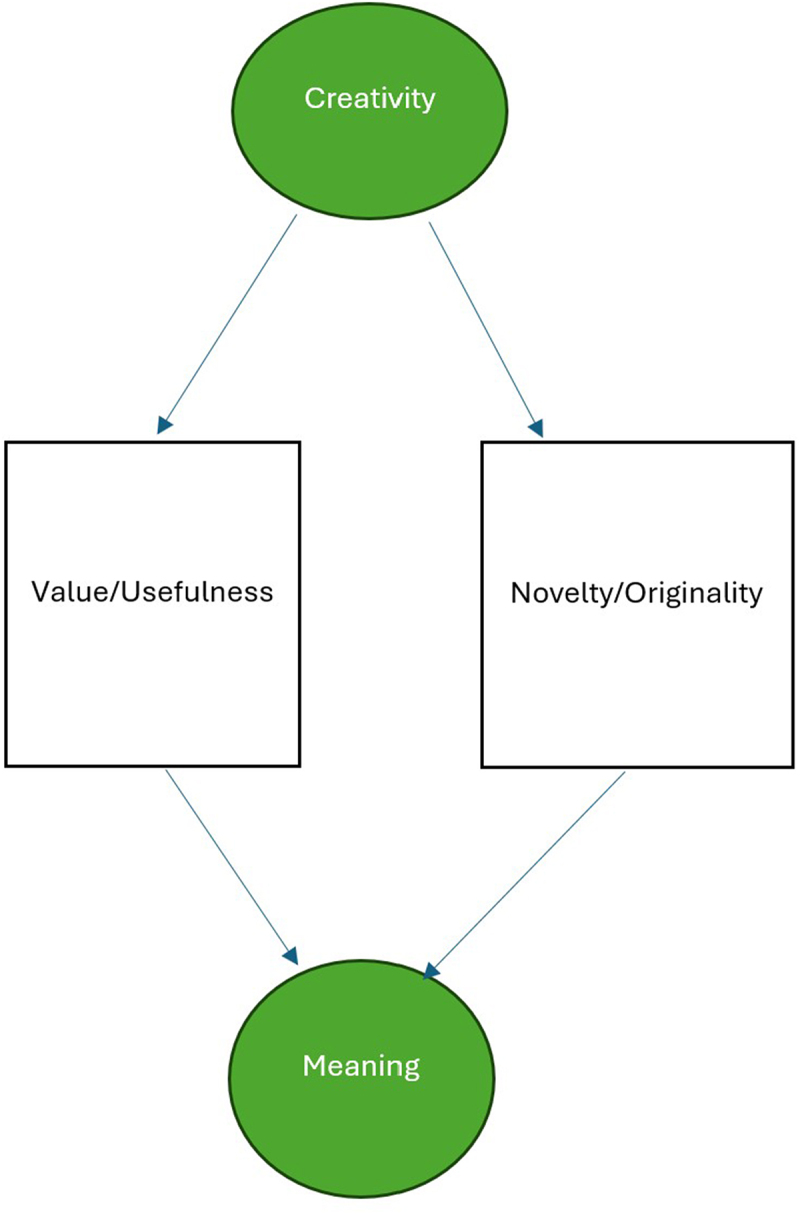
Creativity and meaning in life.

Creative contributions are a critical source of meaning. Indeed, meaning is an essential component of creativity alongside value and novelty [37] (Figure 3). Many of the critical concepts used to define the meaning of life, such as the need for significance, growth, coherence, purpose, or the aspiration for symbolic immortality, can be achieved only through creativity [38–40]. Creativity is is positively correlated with meaning in life, self efficacy and happiness [41,42]. Evidence suggests that creative activity that involves flow decreases suicide risks due to the mediation of purpose in life that it promotes [43]. Meaning from creative activity played a mitigating role against the negative effects of the COVID-19 pandemic [44].

### Creativity, evolution, and essence of existence

From an evolutionary perspective, success is the ability of a population to adapt well to their environment and survive long enough to pass on their genetic material to their offspring. It is measured by population abundance and geographical range [45]. Going by this understanding, humans are a very successful species. Human creativity is the reason for its success. Indeed, human civilization as we know it exists because of the creativity of our ancestors [4,21]. From culture and medicine to technology, all that we enjoy is built on the backs of the creativity of the people who came before us. As members of the human species, we owe an evolutionary or existential debt to our ancestors for their valued and novel contributions to the species’ thriving and must repay that debt by contributing value and novelty that enhances the continued thriving of the species. So, the meaning or essence of human existence from an evolutionary standpoint is to repay our existential debt by engaging in positive creativity. Indeed, creative activity has been described as a virtue [46,47]. This entails that the essence of human life is to work toward human continued thriving and the prevention of human extinction by contributing creatively (“value and novelty”). So, an entrepreneur who leverages their imagination to invent a product that consumers find useful is engaged in a positive creative activity that is in line with repaying their existential debt. In contrast, a scammer or thief who leverages imagination to defraud others is not engaged in positive creativity because while the fraudster may find value in their activity, the victims and their families find no value in it. In endeavors where both value and novelty cannot be provided, the goal must be to provide value. Thus, a healthcare professional who cares for their patients compassionately and ethically is engaging in actions in line with repaying their existential debt.

## The intricate relationship between creativity and meaning

The modern world makes people more vulnerable to meaning crisis. Indeed, meaninglessness has been described as one of the biggest threats of our time [24] and the world it is argued, is in the midst of an existential crisis [26]. There is a rising rate of mental health disorders [48–53] and suicide [54–56] believed to be linked to a crisis of meaninglessness [57]. Many features of the modern world, including the glorification of materialistic values, spiritual emptiness, a culture of fear, erosion of trust, etc., make people more worried, stressed, and vulnerable to meaning crisis [25,58]. A persistent state of heightened anxiety and fear attributable to political polarization, censorship (cancel culture) [59–61], constant exposure to negative news, social media anxieties, economic instability and fustrations contribute to the vulnerability to meaning crisis [25,58,62]. Also, loneliness, a common feature of modern societies [63] is linked with meaning issues [64] and may be contributing to the increased vulnerability to meaning crisis [25]. Many societies are dealing with an erosion of trust or low trust in social, political, economic, and media institutions [65,66] and the rise of populism and misinformation has negatively impacted trust [67,68]. Low trust is associated with increased vulnerability to mental health problems [69–72] and may negatively impact purpose and satisfaction in life [73]. Also, stress is a common feature of modern societies [74–76], that makes people more vulnerable to meaning crisis [25] and perceived stress negatively impacts the meaningfulness of life [77,78].

Importantly, features in the modern world that are implicated in making people vulnerable to meaning crisis also impact creativity, suggesting an interesting relationship between meaning in life and creativity. Indeed, many societies are dealing with low trust [65], and low trust may negatively impact purpose and satisfaction in life [73] as well as creativity [79]. A culture of fear is noted as one of the features of the modern world that increases vulnerability to meaning crisis [25,62] and threats result in reduced activation of the prefrontal cortex and supramarginal gyrus, and increase negative emotions, negatively impact creativity [80]. Also, research indicates that strife and sociocultural pathologies can negatively impact creativity [81] and a culture of fear of risk-taking negatively impacts creativity [82]. Stress is a common feature of modern societies [74–76] that makes people more vulnerable to meaning crisis [25]. It is the driver of the negative effects of many of the features of modern societies that make people more vulnerable to meaning crisis including loneliness, spiritual emptiness, and materialistic pursuit. Low levels of stress may promote creativity [83], but extreme levels negatively impact creativity [84–86]. Indeed, acute stress inhibits creative performance and indirectly negatively impacts individual creativity via the mediating role of cognitive flexibility and cortisol levels [82].

Taken together, the interrelated factors of acute or high levels of stress, low trust, and a culture of fear appear to be the factors in modern society making people vulnerable to meaning crisis and they may negatively impact creativity.

## Discussion

For anything to be defined as creative, it must provide value and novelty. The creative contribution of “positive value and novelty” is vital to human thriving and survival. All that we enjoy in this world is built on the creativity of our ancestors. Therefore, the essence of human existence is to build on what they have done to leave a better world for future generations. Positive creative contributions are the means to repay our existential debt to our ancestors and find meaning in a world full of uncertainty and adversity. So, the question we must ask ourselves daily or periodically to live meaningfully is how can I contribute value or value and novelty to my community or humanity? What contributions am I making to my community, what positive value and novelty am I adding to the world, and what value and novelty will I have added when I die?” A life of crime, corruption, or engagement in activities that cause harm to others is failing in its purpose. Similarly, a mediocre life falls short of an individual’s potential and shortchanges society from the totality of what such an individual can contribute.

The features of the modern world promote the interrelated factors of acute or high levels of stress, low trust, and a culture of fear, and these features may negatively impact mental health, creativity, and meaning, indicating a direct relationship between the trio. High or acute stress is the driver of the negative effects of many of the features of modern societies such as academic stress, work stress, loneliness, spiritual emptiness, and materialistic pursuit make people vulnerable to meaning crisis. Students deal with a great deal of academic stress (from academic performance, high-grade achievement, preparation for tests, expectations from self, teachers, and parents, etc.) that may impact their mental health [87–89], negatively impact creativity [83,90], meaning [91], and risk of suicide/suicide ideation [92–94]. Job stress is a key feature of modern work life [95], and it is associated with negative outcomes [96]. Burnout due to work stress is linked with existential meaninglessness [97]. Also, most jobs involve long hours and are at-will with cuts happening anytime. In a global economic environment, production can be easily moved to other parts of the world where it makes more economic sense and events in one part of the world can impact the job situation of people in another part of the world. This unpredictable job security environment exerts a huge stress toll on many workers and can negatively impact their creativity. Furthermore, materialistic values are linked with significant stress which negatively impacts life satisfaction [98], meaning in life [99], well-being [100] and flow experience and creativity [101–103]. Loneliness is another common feature of modern societies [63], linked with acute stress [104], meaning crisis [34,64], and it has an inverse relationship with creativity [105]. Notably, creativity is a resource against loneliness [106,107]. Moreover, spiritual emptiness is noted as one of the features of modern society that make people vulnerable to meaning crisis [25,58], and evidence suggests it is associated with stress and psychological well-being [108,109]. Creativity and spirituality are inseparable and intricately interwoven [110,111], and it can provide spiritual coping and resilience against adversity [112,113].

To promote psychological well-being and meaningful lives in society, efforts must be made to foster creativity. In this effort, more must be done to allow the free expression of ideas no matter how unpalatable. A careful balance must be struck between protecting individual rights and the freedom of unpalatable speech so as not to create an environment of censorship, fear, and self-curtailed expression and ideas, which may stifle creativity. More must be done at the family, organizational, and societal levels to reduce stress. Reducing the working day to four instead of five could help relieve employee stress and give them some free time to relax which may improve their creative contributions. Increased inclusion of all employees in critical organizational decision-making and having employee job satisfaction and inclusion as organizational goals can increase employees’ sense of workplace value contribution, which in turn can increase their creative output. Organizational culture must also endeavor to enhance employees’ sense of value creation and difference-making to the community or world by creating an environment where all employees, especially junior employees, can innovate or propose ideas that contribute to organizational success. This will enhance employees’ sense of living meaningfully. Public health policy should promote increased creative activities, including arts, dancing, music, sports, volunteering, youth movements [114], and intergenerational initiatives that can help promote social interaction, provide cognitive stimulation, enhance a sense of self-worth, and improve mental health [115]. Campaigns to promote positive creative contributions as the essence of existence, the path to living meaningfully, and the means to repay our existential debt will help increase creative pursuits in society. More government support should be given to people living on the margins to reduce the acute stress from poverty. Consideration should be given to expanding natural environments in urban areas as it may increase creativity [116] and reduce materialistic desires [117].

Furthermore, efforts must be made to make education enjoyable and not just to pass, which promotes academic stress for students. The culture of excessive focus on grades, accolades for academic performance, and graduation celebration reinforce the preeminence of grades, testing, and passing, which increases students’ stress. Lowering standards is unhelpful as it may result in disengaged and apathetic students. The goal should be to elevate the place of creativity in the curriculum and make tangible creative achievement the bar for awards and celebrations. The fact that graduation or receiving good grades warrants celebration shows that parents, students, and even teachers do not understand the place of creativity in society’s progress and its meaning in students’ lives. Passing a course or graduating is evidence that the student has mastered the concepts presented, nothing more. It is not noteworthy or worthy of celebration. If we are to celebrate students who master the ideas of others, what are we supposed to give to the authors who wrote the textbook or whose ideas and works are foundational to the curriculum? The bar for celebration should not be passing or getting excellent grades but eminent creative achievement. Such a low bar for celebration does not inspire young people to achieve greatness (eminent creative achievement). Students must see their education not simply as an exercise in rote learning and memorization, passing required courses, earning excellent grades, and graduating, but as an opportunity to ask questions about the nature of things and to discover how they can work toward solving the most pressing societal problems. Education must be more than the means to have a great career and acquire material things; it must be the vehicle to pursue the immaterial, achieve the enduring, and change the world for the better. Education must imbue in students a creativity-driven life purpose, which is to focus on changing, redirecting, or augmenting some aspects of culture for the better [118]. If the educational system cannot produce creative thinkers, it is failing in its mission. The basic task of education must be to lead students to think critically, support their individuality and creativity, and work with high adaptability and flexibility [119]. It must be able to inspire young people to aspire for greatness and make a significant difference in both their community and the wider world. The government and educators must improve the curriculum to equally include learning core content and completing complex projects that enable students to think critically and creatively. Training and nurturing have been shown to enhance creativity [120–122] and more effort should be made to promote creativity both in educational and organizational settings and in the broader society. In a multicultural modern society where people must live side by side with others unlike themselves, increased educational focus on creativity can be used to reduce conflict as it helps overcome conflict-related biases [123], helps people tolerate ambiguity [124], and reduces prejudice [125].

In conclusion, people tend to reject creative ideas, even when expressing creativity is the desired goal in efforts to reduce uncertainty [126], indicating that creative contributions face significant barriers from human nature, and more must be done to create the right nurturing culture for creativity. Indeed, culture is critical to creativity, and creativity is important to cultural evolution [4,22,127]. Evidence suggests a complex and important interaction between creativity and culture in which both impact each other [128] and culture and environment can inhibit creative activities [129,130]. Creativity is a critical source of meaning and many features of the modern world that promote meaning crisis may also negatively impact creativity, indicating a relationship between creativity and meaning in which meaninglessness may negatively impact creativity and vice versa. To promote psychological well being and ensure people live meaningfully, more must be done to increase creativity in society. Of all creative contributions, eminent original and valued contributions are the most consequential to human progress. To repay our existential debt, the goal of living must be not just to contribute a little bit but to make eminent original and valued contributions that advances human progress. In endeavors where both value and novelty cannot be provided, the goal must be to provide value. Finally, it is important to note that although some evidence suggests that materialism negatively impacts flow experience and creativity [101–103], it has also been linked with openness to experience [131] and entrepreneurship [132].

## Data Availability

Data sharing is not applicable to this article as no datasets were generated or analyzed during the current study.
